# Transcriptome Sequencing-Based Mining of Genes Associated With Pubertal Initiation in Dolang Sheep

**DOI:** 10.3389/fgene.2022.818810

**Published:** 2022-03-03

**Authors:** Zhishuai Zhang, Zhiyuan Sui, Jihu Zhang, Qingjin Li, Yongjie Zhang, Feng Xing

**Affiliations:** Key Laboratory of Tarim Animal Husbandry Science and Technology, Xinjang Production and Construction Group, College of Animal Science, Tarim University, Alaer, China

**Keywords:** Dolang sheep, RNA-seq, puberty initiation, hypothalamus, differential gene expression

## Abstract

Improving the fertility of sheep is an important goal in sheep breeding as it greatly increases the productivity. Dolang sheep is a typical representative breed of lamb in Xinjiang and is the main local sheep breed and meat source in the region. To explore the genes associated with the initiation of puberty in Dolang sheep, the hypothalamic tissues of Dolang sheep prepubertal, pubertal, and postpubertal periods were collected for RNA-seq analysis on the Illumina platform, generating 64.08 Gb clean reads. A total of 575, 166, and 648 differentially expressed genes (DEGs) were detected in prepuberty_vs._puberty, postpuberty_vs._prepuberty, and postpuberty_vs._puberty analyses, respectively. Based on Gene Ontology (GO) and Kyoto Encyclopedia of Genes and Genomes (KEGG) analyses, the related genes involved in the initiation of puberty in Dolang sheep were mined. Ten genes that have direct or indirect functions in the initiation of puberty in Dolang sheep were screened using the GO and KEGG results. Additionally, quantitative real-time PCR was used to verify the reliability of the RNA-Seq data. This study provided a new approach for revealing the mechanism of puberty initiation in sheep and provided a theoretical basis and candidate genes for the breeding of early-pubertal sheep by molecular techniques, and at the same time, it is also beneficial for the protection, development, and utilization of the fine genetic resources of Xinjiang local sheep.

## 1 Introduction

Puberty refers to the age at which an animal first appears to ovulate in heat, indicating an ability to reproduce (Xing et al., 2019). Puberty is influenced by a variety of factors, including genetic mechanisms, nutritional levels, and light hours (Ortavant et al., 1985; Suttie et al., 1985; Greives et al., 2007). The gonadostat hypothesis suggests that as the body develops, hypothalamic *GnRH* neurons become less sensitive to the negative feedback effects of steroid hormones, while pulsatile *GnRH* secretion increases, stimulating gonadotropin secretion and ultimately leading to follicle development and ovulation (Day & Anderson 1998).

Puberty is a physiological phenomenon caused by the development of follicles in the ovaries, and it is regulated by the hypothalamic–pituitary–ovarian axis. Normal or disturbed pubertal development is mainly determined by genetic factors (Roth and Ojeda 2005; Gajdos et al., 2010). Several prior studies have shown that important hypothalamic regulatory gene systems in the initiation of puberty include the leptin system (Hausman et al., 2012), the neurohormone B system (Tusset et al., 2012), the γ-aminobutyric acid system (Terasawa & Fernandez 2001), the Lin28 system (Tommiska et al., 2010), and the microRNA system.

Dolang sheep is the most common sheep breed in the southern region of Xinjiang, known for their high litter rate, adaptability, and perennial heat (Chang et al., 2020). The regulatory mechanism of puberty in Dolang sheep is still unclear, and gene regulation in this regard has not been sufficiently studied in Dolang sheep. Transcriptome sequencing analysis is widely used to screen differential genes, identify candidate genes, analyze metabolic pathways, and predict the relationship between genes and target organs (Marguerat and Bahler 2010; Chen et al., 2011; Ramayo-Caldas et al., 2012). Gao et al. discovered the candidate lncRNA *XLOC_446,331*, which may play a crucial role in regulating female puberty by transcriptome sequencing (Gao et al., 2018). Similarly, Li et al. (2021) identified six (*ESR1*, *NF1*, *APP*, *ENPP2*, *ARNT*, and *DICER1*) genes associated with proestrus by performing differential RNA-seq analysis of hypothalamic tissue in sows’ prepuberty, during puberty, and postpuberty. Ling et al. (2014) identified 12 genes associated with high fecundity in Anhui white goats. Based on metabolomics, Zhou et al. (2021) identified polymorphisms in IRS1 were associated with growth efficiency traits in Chinese black Tibetan sheep and Zhang et al. found stall-feeding modified the content of protein and fat, tenderness, water holding capacity, and texture of the longissimus lumborum of Tibetan sheep (Zhang et al., 2021). Based on 16S rRNA gene pyrosequencing, Gui et al. (2021) found that supplementation of concentrate in the cold season improved the rumen microbial abundance of Tibetan sheep. Thus, the biological processes of an organism can be studied in depth using transcriptomics (Conrad et al., 2018). We used transcriptomic and bioinformatics analyses to identify differentially expressed genes in the hypothalamus of Dolang sheep during different periods of puberty and tried to identify candidate genes that might be associated with puberty in Dolang sheep.

## 2 Materials and Methods

### 2.1 Materials and Treatments

Dolang sheep maintained in the Tarim University experimental station were used as the model in this study. The ewes were observed at 10 o ‘clock, 14 o ‘clock, and 18 o ‘clock every day. The criteria for judging puberty in ewes were mental restlessness, the tendency to walk, the acceptance of ram riding, and the presence of mucus in the vulva. All sheep were in good health. Hypothalamus samples were collected from ewes that were first found to be undergoing puberty, immediately frozen in liquid nitrogen, and stored at −80°C for further analysis. The hypothalami were further collected from prepubertal and postpubertal ewes maintained in the same manner. A total of nine sheep’s hypothalamic tissues were collected. Three biological replicates were performed for each period and analyzed.

### 2.2 Nucleic Acid Extraction and RNA-Seq Library Construction

The total RNA content from the hypothalami was extracted using TRIzol reagent (Beijing Kangwei Century Biotechnology Co.). The purity, concentration, and integrity of the RNA samples were tested by Nanodrop (Thermo Fisher, United States) and Agilent 2100 (Agilent Technologies, United States). A total of 1 μg RNA per sample was used as the input material for the RNA sample preparations. mRNA was purified from total RNA using poly-T oligo-attached magnetic beads. Fragmentation was carried out using divalent cations under elevated temperature in NEBNext First Strand Synthesis Reaction Buffer (5X). First strand cDNA was synthesized using a random hexamer primer and M-MuLV reverse transcriptase. Second strand cDNA synthesis was subsequently performed using DNA polymerase I and RNase H. Remaining overhangs were converted into blunt ends *via* exonuclease/polymerase activities. After adenylation of 3′ ends of DNA fragments, NEBNext adapter with a hairpin loop structure were ligated to prepare for hybridization. In order to select cDNA fragments of preferentially 240 bp in length, the library fragments were purified with the AMPure XP system (Beckman Coulter, Beverly, United States). Then, 3 μl USER enzyme (NEB, United States) was used with size-selected, adapter-ligated cDNA at 37°C for 15 min followed by 5 min at 95°C before PCR. Then, PCR was performed with Phusion High-Fidelity DNA polymerase, Universal PCR primers, and index (X) primer. At last, PCR products were purified (AMPure XP system), and library quality was assessed using the Agilent Bioanalyzer 2100 system. Clustering of the index-coded samples was performed on a cBot Cluster Generation System using TruSeq PE Cluster Kit v4-cBot-HS (Illumina) according to the manufacturer’s instructions. After cluster generation, the library preparations were sequenced on an Illumina platform (Biomarker Technologies), and paired-end reads were generated.

### 2.3 Sequencing Data Analysis

In the present study, transcript libraries from Dolang sheep at the developmental stages of prepuberty, puberty, and postpuberty were constructed and assayed by high-throughput RNA-seq. Clean reads were obtained using the program Trimmomatic v0.32 (Bolger et al., 2014) by removing reads containing adapters, reads containing poly-N, and low-quality raw reads. Clean reads were then aligned and mapped to the sheep genome (Oar_v4.0) using HISAT2 (Kim et al., 2015). The matched reads were assembled and gene or transcript expression was calculated using StringTie (Pertea et al., 2015). Gene or transcript expression levels were quantified using fragments per kilobase of transcript per million fragments mapped (FPKM) (Florea et al., 2013).

### 2.4 Sample Correlation Analysis

To assess the reliability of the tested samples, the degrees of variation among the three groups were analyzed using replicate scatter. It was finished by R package corrplot (Wei et al., 2017) and ggplot2 (Wickham 2011).

### 2.5 Identification and Functional Enrichment Analysis of DEGs

Relative gene expression levels among prepuberty, puberty, and postpuberty were counted using the log2 ratio. The differentially expressed genes (DEGs) were identified using the DEseq2 (Love et al., 2014) method, with the cutoff set as a fold change ≥1.5 and *p*-value ≤ 0.05. The number of differentially expressed genes among the three groups was compared. Hierarchical clustering analysis was performed on the screened differentially expressed genes using R package heatmap.2 (Warnes et al., 2016), and the genes with the same or similar expression patterns were clustered.

Gene function was annotated *via* alignment with multiple databases, including the Gene Ontology (GO) and Kyoto Encyclopedia of Genes and Genomes (KEGG) databases.

### 2.6 Soft Cluster Analysis of DEGs

The soft clustering Mfuzzy function is based on the fuzzy k-means algorithm in e1071 package. R package Mfuzz was used to assign a gene to several clusters using soft clustering methods (Kumar and Futschik 2007). This analysis method can identify potential time series patterns of expression profiles and cluster genes with similar patterns. This can reveal the dynamic patterns of genes and how they are functionally connected.

### 2.7 qRT-PCR Validation of RNA-Seq Data

Thirteen genes were selected to verify the RNA-Seq results using quantitative real-time PCR (qRT-PCR). Reverse transcription was performed using the reverse transcription kit (TaKaRa). The 15 μl PCR reaction mixture consisted of 5.5 μl ddH_2_O, 7.5 μl PerfectStart Green qPCR SuperMix (2×), 1 μl cDNA, and 0.5 μl of each primer (10 μM). The thermal cycle parameters were as follows: 95°C for 15 s, 95°C for 15 s, 55°C for 15 s, and 68°C for 20 s for 40 cycles. Three technical replicates were performed for each sample. Actin was used as an internal reference. The relative expression levels of genes were calculated using the 2^−△△CT^ method (Ju et al., 2020). SPSS 17.0 software package (SPSS, Chicago, IL, United States) was applied to analyze the qRT-PCR data.

### 2.8 Ethical Approval

This study was conducted in accordance with the specifications of the Ethics Committee of the Tarim University of Science and Technology.

## 3 Results

### 3.1 Identification of Puberty in Dolang Sheep

**FIGURE 1:**
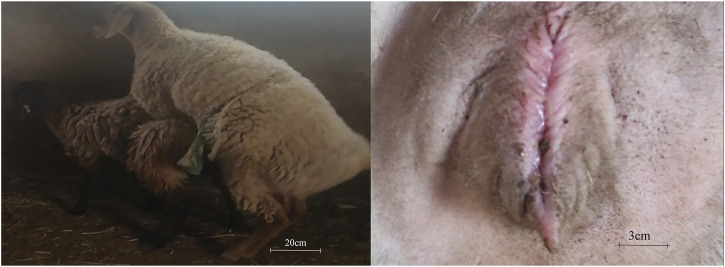
Ewes undergoing puberty were defined as those receptive to climbing by rams and mucus-laden vulva.

Pubertal features of Dolang sheep were observed (Figure 1). At the prepuberty stage of ewe, they are calm and do not accept ram for riding, and the vulva is dry. At the pubertal stage, ewes accept ram riding and a moist external pudenda can be observed. At the end of puberty, the ewes become calm, again refused the climbing of ram, and the vulva becomes dry.

### 3.2 Sequencing Data Quality Control and Sequence Alignment With Reference Genomes

As shown in Table 1, after removing reads containing adapters, reads containing ploy-N, and low-quality reads from raw reads, 64.08 GB clean reads and the percentage of Q30 base of each sample was > 92.05%, with an average GC content of 50.79%. Reads were submitted to the NCBI Sequence Read Archive under the accession number PRJNA773843.

**TABLE 1:** Summary of read numbers of in the prepuberty, puberty, and postpuberty groups.

Sample name	Prepuberty_1	Prepuberty_2	Prepuberty_3	Puberty_1	Puberty_2	Puberty_3	Postpuberty_1	Postpuberty_2	Postpuberty_3
Total clean reads	47,762,312	48,092,774	50,578,284	50,126,846	42,344,556	46,988,672	50,369,858	43,405,702	48,303,884
Clean read q20 (%)	97.14	97.05	97.1	97.04	96.64	97.04	97.09	96.97	97.1
Clean read q30 (%)	92.92	92.73	92.84	92.72	92.05	92.71	92.82	92.57	92.80
Total mapping genome reads	42,408,942	42,929,428	44,954,682	44,971,014	35,823,567	41,661,795	44,724,205	38,712,157	42,397,509
Total mapping genome ratio (%)	88.79	89.26	88.88	89.71	84.60	88.66	88.79	89.19	87.77

The clean reads in each library were then aligned to the sheep genome (Oar_v4.0). The proportion of clean reads mapped to the reference genome ranged from 84.60 to 89.71%, among which 88.74% were uniquely mapped. The percentage of reads mapped to sense strands ranged from 35.09 to 38.63% and those mapped to the antisense strands ranged from 39.35 to 41.95%.

### 3.3 Quantification of Gene Expression Levels and Correlation Assessment of Biological Replicates

**FIGURE 2:**
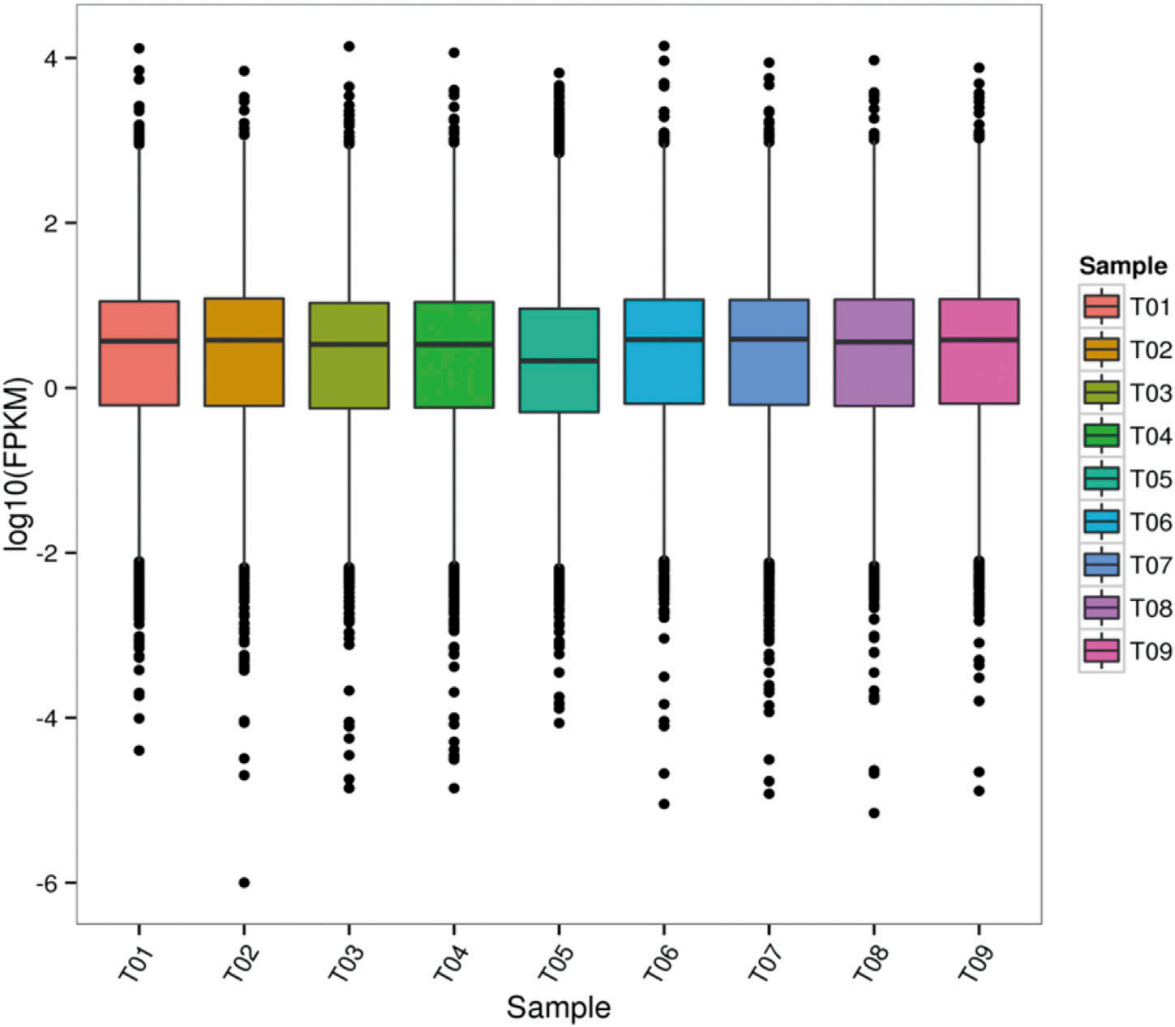
FPKM box line diagram for each sample. T01, T02, and T03 were the prepubertal groups. T04, T05, and T06 were the pubertal groups. T07, T08, and T09 were the postpubertal groups.

Gene expression levels were estimated by fragments per kilobase of transcript per million fragments mapped. $FPKM = {cDNA fragments \over { mapped fragments (million) × transcript length (kb)}}$. We used StringTie software to evaluate gene expression levels (Figure 2).

**FIGURE 3:**
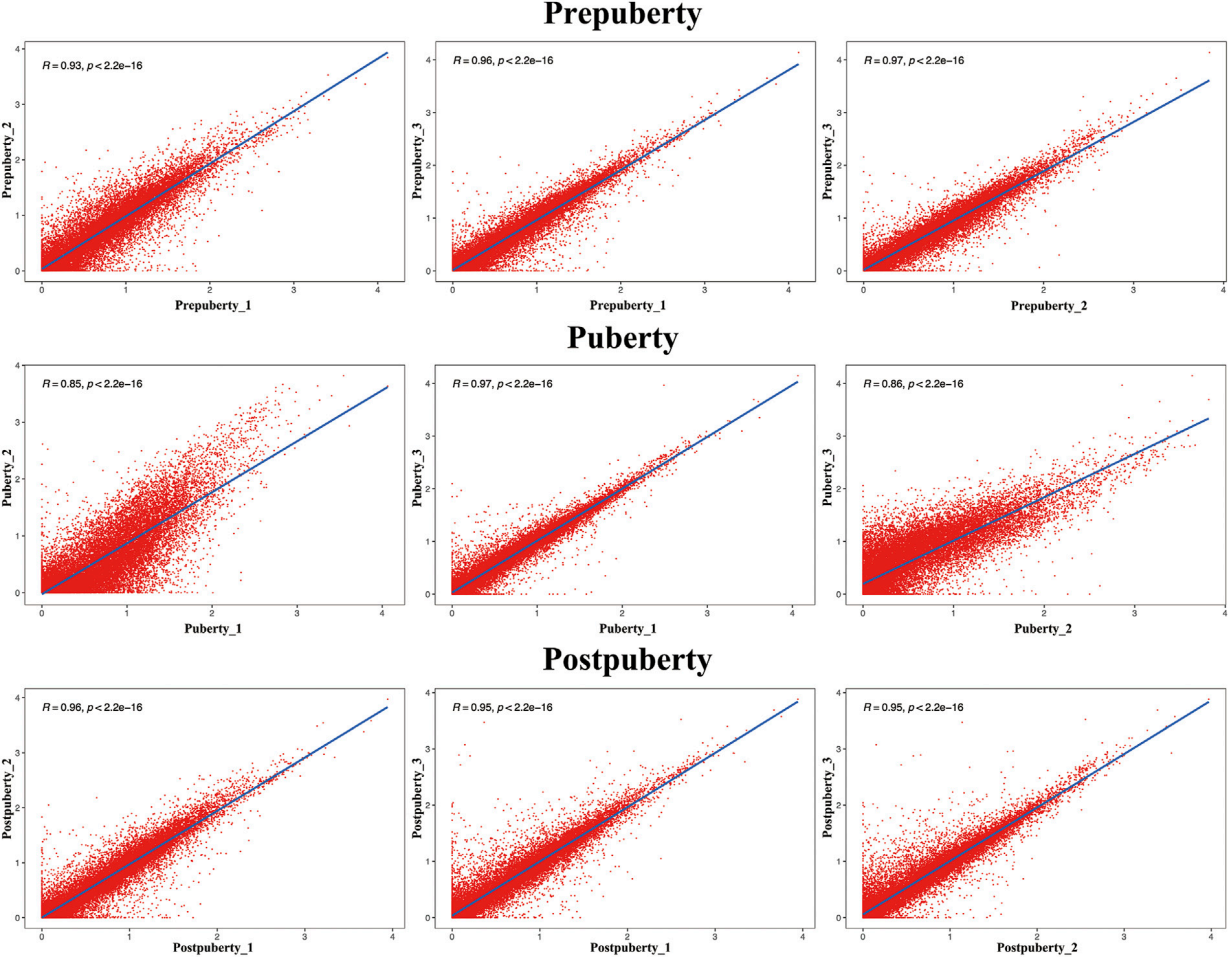
Assessment of correlations among the three groups by analysis of replicate scatter.

Biological repeat correlation was calculated using R package of the corrplot (Figure 3). In the prepuberty, puberty, and postpuberty groups, correlation coefficients among samples were greater than 0.93, 0.85, and 0.95, respectively. These results suggest that the sampling of hypothalamus in the present experiment is reliable and suitable for further analysis.

### 3.4 Identification of Differentially Expressed Genes Among Prepuberty, Puberty, and Postpuberty Ewes

**FIGURE 4:**
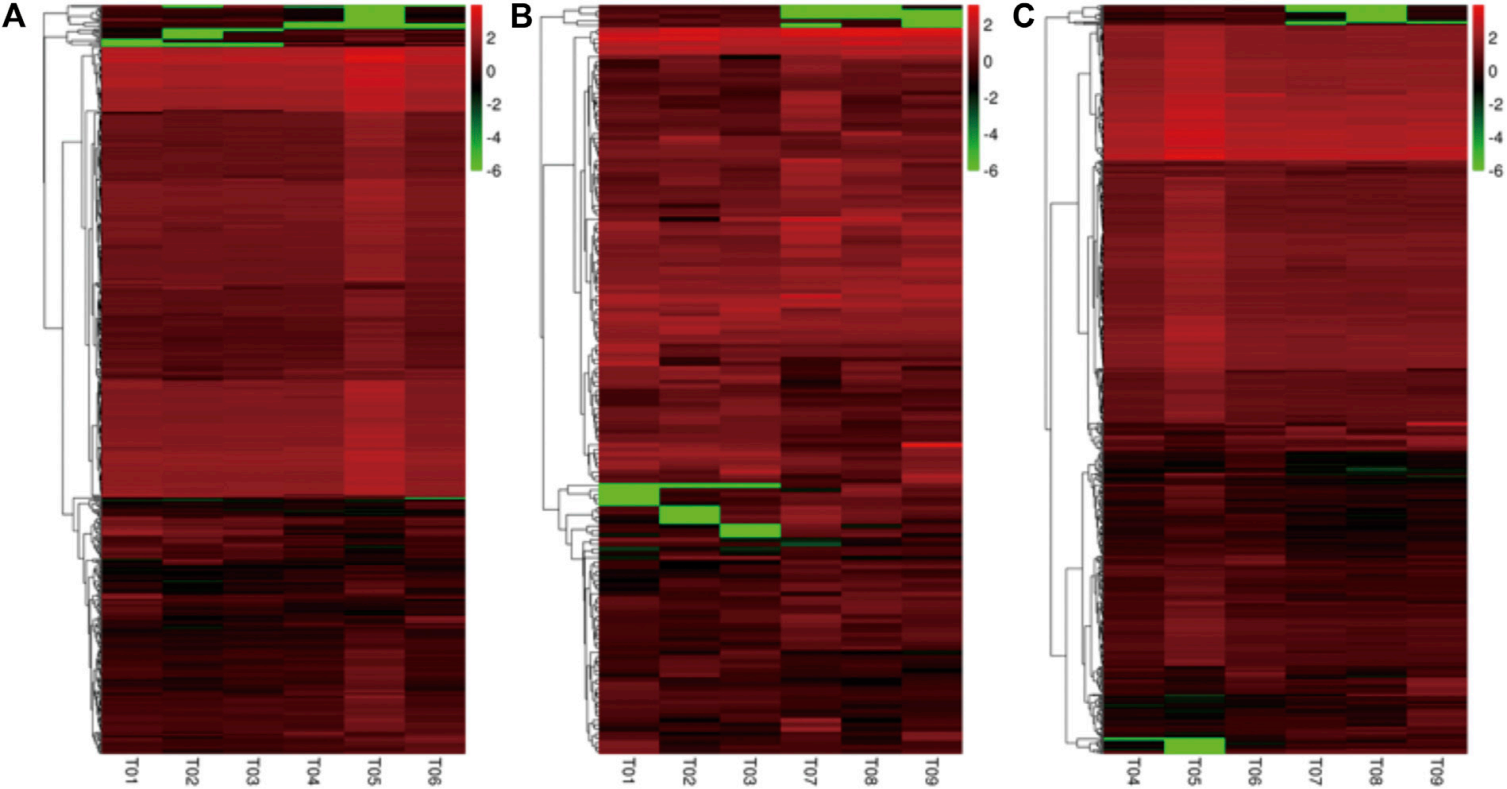
Cluster analysis of all differentially expressed genes. **(A)** Differentially expressed genes between the prepuberty (T01, T02, and T03) and puberty (T04, T05, and T06) groups. **(B)** Differentially expressed genes between the prepuberty (T01, T02, and T03) and postpuberty (T07, T08, and T09) groups. **(C)** Differentially expressed genes between the puberty (T04, T05, and T06) and postpuberty (T07, T08, and T09) groups.

**FIGURE 5:**
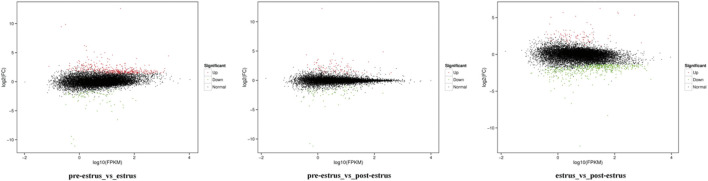
Expression profiles of the identified DEGs. Red and green points represent significant DEGs with *p*-value ≤ 0.05, and fold change ≥1.5, and black points represent those without significance.

Based on the FPKM method, the transcript abundance of each gene from prepuberty, puberty, and postpuberty data was analyzed (Figures 4, 5). In the comparison of prepuberty and puberty ewes, 575 genes exhibited a significant difference in their expression levels with a threshold of *p*-value ≤ 0.05 and fold change ≥ 1.5, including 490 upregulated and 85 downregulated genes. In the comparison of prepuberty and postpuberty, 166 genes exhibited a significant difference in their expression levels with the threshold of *p*-value ≤ 0.05, and fold change ≥ 1.5, including 96 upregulated and 70 downregulated genes. In the comparison of puberty and postpuberty, 648 genes exhibited a significant difference in their expression levels with the threshold of *p*-value ≤ 0.05 and log_2_ (fold change) ≥ 1.5, including 97 upregulated and 551 downregulated genes (Table 2).

**TABLE 2:** The number distribution of differentially expressed genes in different sample group.

DEG set	DEG number	Upregulated	Downregulated
Prepuberty_vs._Puberty	575	490	85
Prepuberty_vs._Postpuberty	166	96	70
Puberty_vs._Postpuberty	648	97	551

### 3.5 Functional Enrichment Analysis of DEGs

#### 3.5.1 DEGs Between Prepuberty and Puberty

**FIGURE 6:**
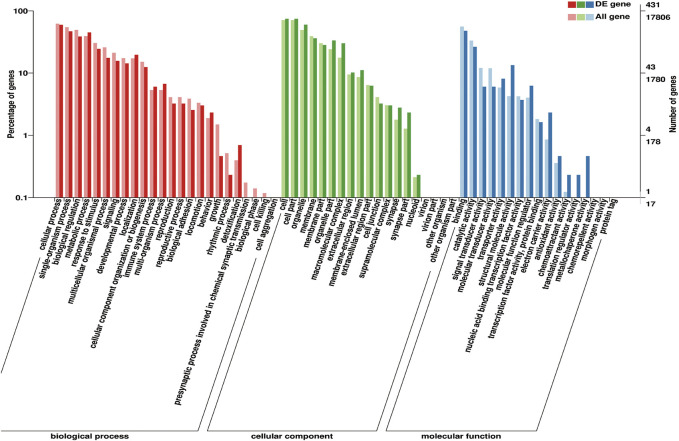
Gene Ontology (GO) classification of 575 DEGs. GO terms are summarized in three main categories: cellular components, molecular functions, and biological processes.

These identified DEGs were annotated with 20 biological processes, 15 cellular components, and 14 molecular functions in the GO categories (Figure 6). The binding and catalytic activities were the top two terms in the molecular function category. In the cellular component category, DEGs were mainly distributed in terms of cell, cell part, and organelle. The most abundant terms in the biological process category were cellular processes and single-organism processes. Reproduction and reproductive processes ranked 13th and 14th, respectively, in the biological process. KEGG pathway analysis classified the DEGs into 199 metabolic pathways.

GO terms associated with puberty were found, including *“*response to estrogen” (GO: 0043627), “cellular response to gonadotropin stimulus” (GO: 0071371), “copulation” (GO: 0007620), “developmental process involved in reproduction” (GO: 0003006), “female pregnancy” (GO: 0007565), “estrogen receptor binding” (GO: 0030331), and “ovarian follicle development” (GO: 0001541). Some pathways related to puberty were made out, exempli gratia, “estrogen-signaling pathway” (ko04915), “oxytocin-signaling pathway” (ko04921), “GnRH-signaling pathway” (ko04912), and “progesterone-mediated oocyte maturation” (ko04914).

During the progression of prepuberty to puberty, *StAR* expression is upregulated and promotes cholesterol metabolism to pregnenolone in the ovarian steroidogenesis pathway. *GIRK* (G protein-gated inwardly rectifying potassium) showed higher expression levels in the estrogen-signaling pathway in prepuberty than in puberty. Upregulation of the *SOHLH1* and *GAMT* genes was found in ovarian follicle development and reproduction, respectively. *APC/C* (anaphase-promoting complex/cyclosome), *MYT1* (myelin transcription factor 1), and *MAPK* (mitogen-activated protein kinase) were upregulated during progesterone-mediated oocyte maturation. The prolactin signaling pathway members *P38* and *IRF-1* (interferon regulatory factor-1) were upregulated and downregulated, respectively.

#### 3.5.2 DEGs Between Prepuberty and Postpuberty

**FIGURE 7:**
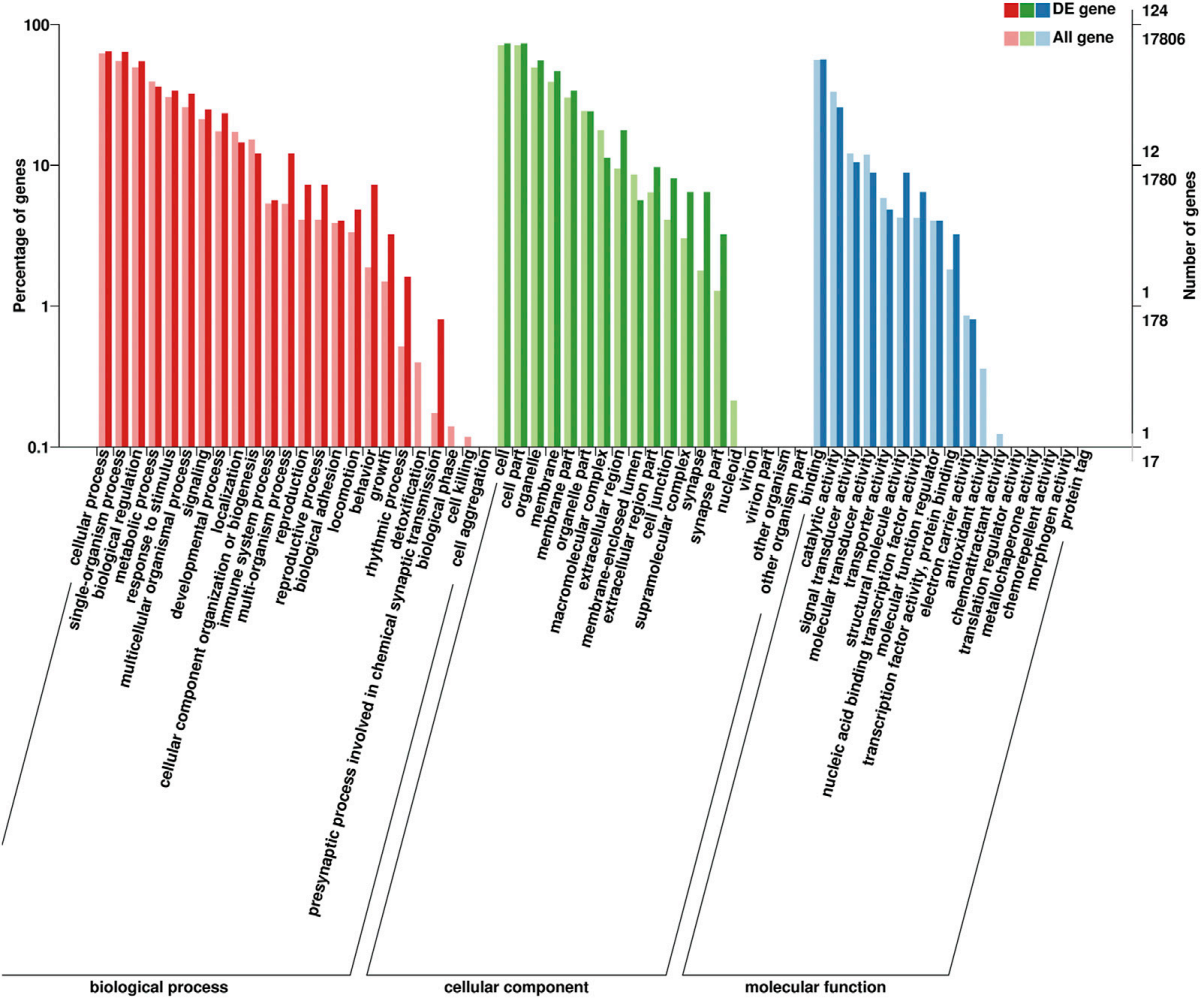
Gene Ontology (GO) classification of 166 DEGs. GO terms are summarized in three main categories: cellular components, molecular functions, and biological processes.

Identified DEGs were annotated with 20 biological processes, 14 cellular components, and 10 molecular functions in the GO categories (Figure 7). The binding and catalytic activities were the top two terms in the molecular function category. In the cellular component category, DEGs were mainly distributed in terms of cell, cell part, and organelle. The most abundant terms in the biological process category were cellular processes and single-organism processes. Reproduction and reproductive processes ranked 13th and 14th in the biological process, respectively. The KEGG pathway analysis classified the DEGs into 94 metabolic pathways.

GO terms associated with puberty including *“*copulation” (GO: 0007620), “ovarian follicle development” (GO: 0001541), “mating behavior” (GO: 0007617), “female pregnancy” (GO: 0007565), “fertilization” (GO: 0009566), “oocyte maturation” (GO: 0001556), and “cell differentiation involved in embryonic placenta development” (GO: 0060706) were included. Several pathways related to puberty, *“*oxytocin-signaling pathway” (ko04921) and “prolactin-signaling pathway” (ko04917) were further identified as differentially regulated.

As ewes progress from puberty to postpuberty, downregulation of *p38MAPK* leads to a reduction in the extent of its effect on gonadotropin gene expression and secretion. Upregulation of the *DMC1* gene has been observed in ovarian follicle development. *StAR* was downregulated and returned to the same level as in prepuberty ewes. In the oxytocin-signaling pathway, protein kinase C (PKC) and *GIRK* are upregulated. *p38MAPK* (p38 mitogen-activated protein kinase) is downregulated in the GnRH-signaling pathway. In the prolactin-signaling pathway, *p38* was downregulated, and *TH* and *IRF-1* (interferon regulatory factor 1) were upregulated. In progesterone-mediated oocyte maturation, *MAPK*, *Myt1*, and *APC/C* were downregulated.

#### 3.5.3 DEGs Between Puberty and Postpuberty

**FIGURE 8:**
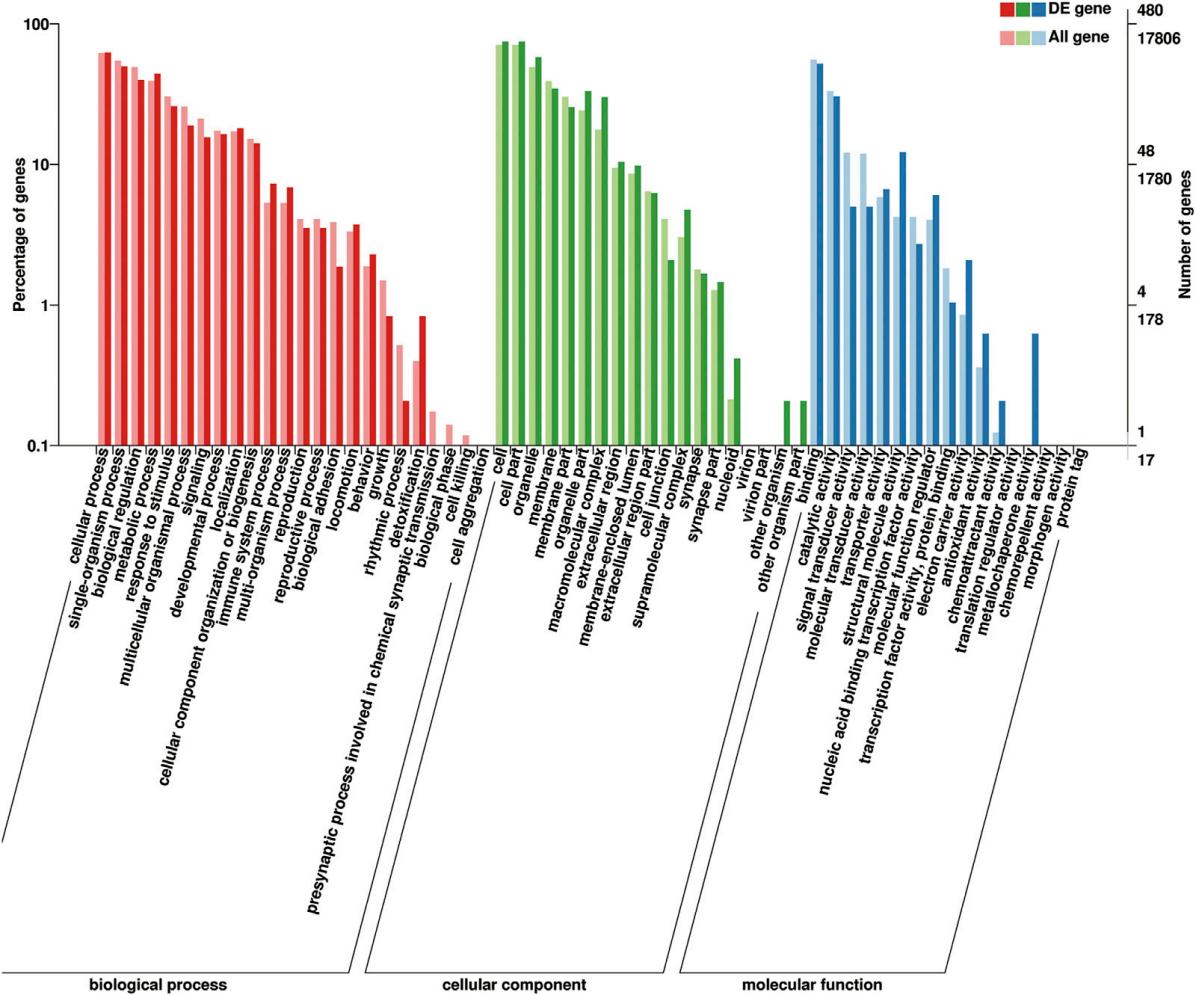
Gene Ontology (GO) classification of 648 DEGs. GO terms are summarized in three main categories: cellular components, molecular functions, and biological processes.

These identified DEGs were annotated in 20 biological processes, 17 cellular components, and 13 molecular functions in the GO categories (Figure 8). The binding and catalytic activities were the top two terms in the molecular function category. In the cellular component category, DEGs were mainly distributed in terms of cell, cell part, and organelle. The most abundant terms in the biological process category were cellular processes and single-organism processes. Reproduction and reproductive processes ranked 13th and 14th, respectively, of the biological process. The KEGG pathway analysis classified the DEGs into 213 metabolic pathways.

GO terms associated with puberty were found, including “post-embryonic development” (GO: 0009791), “developmental process involved in reproduction” (GO: 0003006), “gonad development” (GO: 0008406), “copulation” (GO: 0007620), “fertilization” (GO: 0009566), “mating behavior” (GO: 0007617), “positive regulation of germinal center formation” (GO: 0002636), and “estrogen receptor binding” (GO: 0030331). Some pathways related to puberty were made out, exempli gratia, “ovarian steroidogenesis” (ko04913), “estrogen-signaling pathway” (ko04915), “oxytocin-signaling pathway” (ko04921), “progesterone-mediated oocyte maturation” (ko04914), and “GnRH-signaling pathway” (ko04912).

During the process of ewe development from prepuberty to postpuberty, calmodulin-dependent protein kinase (CaMKK), and soluble guanylyl cyclase (sGC) were found to be up- and downregulated in the oxytocin signaling pathway, respectively. *PRL* and *TH* were upregulated in the PRL signaling pathway. *TTR* was found to be downregulated in hormone activity entry.

### 3.6 Time Series Expression Clustering Analysis

**FIGURE 9:**
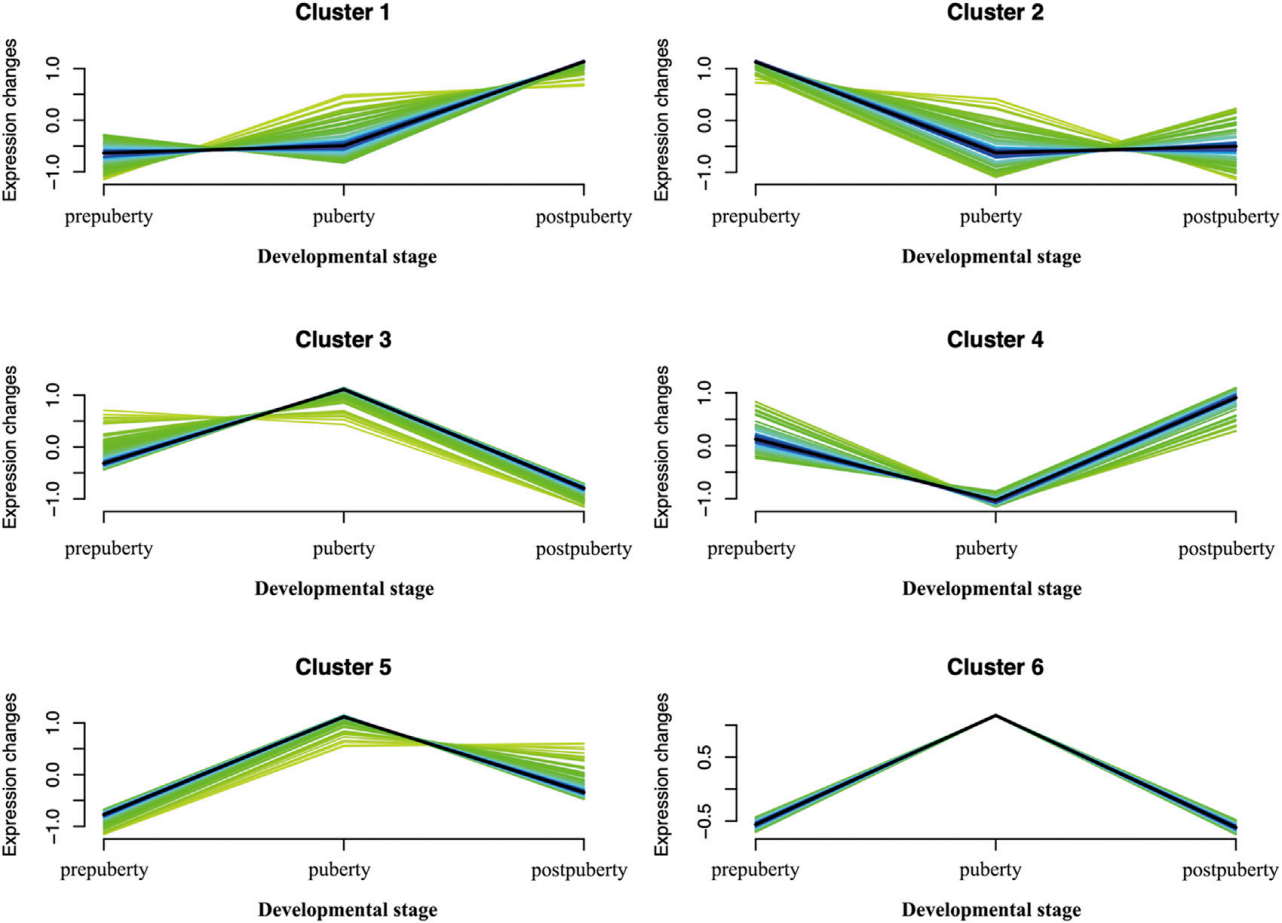
Soft clusters of differently expressed genes. Twelve puberty-related clusters. The horizontal axis represents the different developmental stages (prepuberty, puberty, and postpuberty). The vertical axis represents the changes in expression.

We selected 811 DEGs that were found to draw clustering maps of the time series (Figure 9). By clustering maps of time series and reviewing related literature, we selected the top 10% of genes in each cluster, and identified a portion of genes associated with puberty, for example, the *SPIN1* gene in cluster3, *DMC1* gene in cluster1, *WISP1* genes in cluster2, and *WNT2B* gene in cluster4. All these genes play a direct or indirect role in puberty initiation (Supplementary Table S1).

### 3.7 DEGs Involved in Puberty in Dolang Sheep

Based on these results, a number of genes associated with puberty were identified. The *GAMT* gene plays an important role in embryonic development and the reproductive system (Braissant et al., 2005; Zhao et al., 2021). *SOHLH1* is a multifunctional regulator of the network required for oocyte maintenance and differentiation during early folliculogenesis (Wang et al., 2020). And SOHLH1 is expressed in early oocytes and is necessary for its differentiation (Pangas et al., 2006). *DMC1* is associated with meiosis (Dalman et al., 2019). Loss of function of DMC1 results in defective meiosis and sterility in many species (Chen et al., 2021). The *MACROD1* gene, also known as *LRP16*, plays a role in estrogen signaling (Han et al., 2007; Meng et al., 2007; Tian et al., 2009). It was identified as an estrogen-responsive gene (Han et al., 2003). Estrogen plays a critical role in female puberty and is also important in many aspects of male puberty (Alonso and Rosenfield 2002). *WNT2B* may be regulated during early pregnancy (Atli et al., 2011). Hatzirodos et al. found in the bovine adult ovary that WNT2B is downregulated in the theca interna of large (9–12 mm) compared to small (3–5 mm) healthy follicles (Hatzirodos et al., 2014). *SPIN1* regulates the meiotic cell cycle by modulating the activation of the spindle assembly checkpoint (Choi et al., 2019). *CRH* may play multiple roles in the human endometrium by modulating different signaling cascades (Karteris et al., 2004). *TTR* may be a candidate gene affecting the difference in lambing number in FecB-free mutant small Tail Han sheep (Zhang 2020). *WISP1* plays an important role in embryonic development and immune-related physiological mechanisms (Wu et al., 2012). Co-expression of *WNT2B* and *WISP1* was found by string (https://string-db.org/cgi/input?sessionId=bmuyn8NiPGtp&input_page_show_search=on).

### 3.8 Expression Profile Analysis by RT-qPCR

**FIGURE 10:**
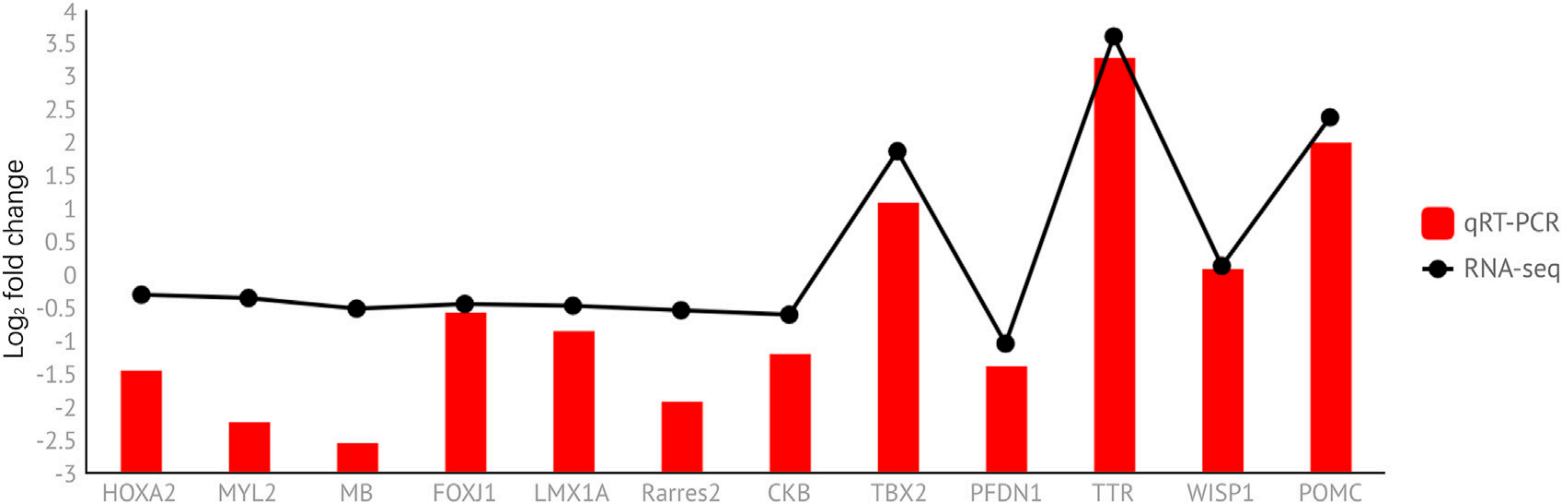
Expression level of DEGs was verified using qRT-PCR and compared with the corresponding data from RNA-Seq assays. The *y*-axis indicates normalized expression levels of the transcripts. The *x*-axis indicates differentially expressed genes.

To verify the accuracy of the transcriptome sequencing results, we selected 12 differential genes (three genes from prepuberty_vs._puberty and nine genes from puberty_vs._postpuberty) to verify by qRT-PCR and calculated the logarithm of the differential fold of gene expression (Figure 10). The correlation coefficients between the two data points were also calculated. The results showed that the correlation coefficient between the RNA-seq and qRT-PCR results was 0.931 (*p* < 0.01). The presence of strong correlations indicates that the RNA-seq results were reliable (Supplementary Table S2).

## 4 Discussion

During the initiation of puberty, secondary sexual characteristics begin to develop, the gonads become mature, and ewes gradually acquire the ability to reproduce. In recent years, significant research has been carried out on the initiation of puberty in animals, and many genes associated with the initiation of puberty in sheep have been identified. *NKB* may be an important component of puberty initiation in sheep (Bedenbaugh et al., 2020). Melatonin has a facilitative effect on the initiation of puberty in ewes (Pool et al., 2020). Mutations in the *BMPR-IB* gene cause earlier initiation of puberty in lambs (Wang 2020). *KISS1* expression in the arcuate nucleus increases during puberty in ewes and may be a causative factor in pubertal activation of the reproductive axis. Furthermore, the decrease in *RFRP* expression may be a factor in the initiation of puberty (Li et al., 2020).

In this study, we filtered, assembled, and compared transcriptome data, screened differential genes, and classified genes by bioinformatics analysis, annotated, and functionally classified genes. As such, we identified several genes related to the initiation of puberty in animals. Several software packages, such as HISAT2, StringTie, and DEseq2 were used.

GO classification of Dolang sheep transcriptome suggests that its properties are related to cellular components, biological processes, and molecular functions. Transcriptome analysis of Dolang sheep using the KEGG database identified the estrogen-signaling pathway, ovarian steroidogenesis, oxytocin-signaling pathway, progesterone-mediated oocyte maturation, prolactin-signaling pathway, and GnRH-signaling pathway. These pathways may be associated with the initiation of initial puberty and reproduction in Dolang sheep. Finally, we successfully identified nine genes (*GAMT*, *SOHLH1*, *DMC1*, *MACROD1*, *WNT2B*, *SPIN1*, *CRH*, *TTR*, and *WISP1*) associated with the initiation of sheep primiparity.

The *GAMT* gene plays an important role in embryonic development (Braissant et al., 2005). In this study, the *GAMT* gene was upregulated in prepuberty_vs._puberty and downregulated in puberty_vs._postpuberty. This indicates that the *GAMT* gene starts to initiate expression during puberty in the preparation for embryonic development after mating. *SOHLH1* is a transcriptional regulator that plays a role in the maintenance and survival of primordial ovarian follicles (Bayram et al., 2015). *SOHLH1* is upregulated in prepuberty_vs._puberty, possibly in preparation for sperm–egg cell binding. *DMC1* plays a role in the initiation and progression of meiosis (Habu et al., 1996). In this study, the expression of *DMC1* increased during the initiation and at the end of puberty in Dolang sheep, indicating that *DMC1* may play a role in promoting oocyte meiosis during the initiation of puberty in Dolang sheep. The *MACROD1* gene is also known as *LRP16*, and *LRP16* plays a role in estrogen signaling (Han et al., 2007; Meng et al., 2007; Tian et al., 2009). In prepuberty_vs._puberty, *MACROD1* gene expression is increased, suggesting that *MACROD1* may function during puberty initiation by influencing estrogenic signaling. *WNT2B* and *WISP1* are co-expressed. *Wnt2B* and *WISP1* are enriched in the Wnt-signaling pathway The Wnt-signaling pathway is expressed in granulosa cells, regulated by gonadotropins, and plays a role in follicle development, ovulation, and luteal formation.

## 5 Conclusion

In terms of mRNA expression, *GAMT*, *SOHLH1*, *DMC1*, *MACROD1*, *WNT2B*, *SPIN1*, *CRH*, *TTR*, and *WISP1* genes were significantly different in their expression in the hypothalamus during different pubertal periods in Dolang sheep, suggesting that these genes may be key genes that directly or indirectly influence the initiation of puberty in Dolang sheep. These results provide a basic theoretical basis for further studies on the molecular mechanisms of pubertal initiation in Dolang sheep.

## Data Availability

The original contributions presented in the study are publicly available. This data can be found here: National Center for Biotechnology Information (NCBI) BioProject database under accession number PRJNA773843.
